# Dynamics of Ras Complexes Observed in Living Cells

**DOI:** 10.3390/s120709411

**Published:** 2012-07-09

**Authors:** Xiangyong Li, Zhiyong Cheng, Honglin Jin

**Affiliations:** 1 Britton Chance Center for Biomedical Photonics, Wuhan National Laboratory for Optoelectronics, Huazhong University of Science and Technology, Wuhan 430074, China; E-Mail: xlxyongli@gmail.com; 2 MoE Key Laboratory for Biomedical Photonics, Department of Biomedical Engineering, Huazhong University of Science and Technology, Wuhan 430074, China; 3 Wuhan Mechanical Technology College, Wuhan 430075, China; E-Mail: zycheng72@sina.com

**Keywords:** K-Ras, Raf1, BiFC, membrane association, signal pathway

## Abstract

K-Ras works as a switch in many important intracellular signaling pathways and plays important roles in cell growth, proliferation, differentiation and carcinogenesis. For signal transduction from K-Ras to Raf1, the best-characterized effector of K-Ras, the general view is that Ras recruits Raf1 from the cytoplasm to the cell membrane. To elucidate this process, we constructed a series of fusion proteins (including Raf1 and K-Ras fused with either fluorescent proteins or fluorescent protein fragments) to compare subcellular localizations of these proteins. Bimolecular fluorescence complementation (BiFC) and a co-transfection system were used. In the BiFC system, the K-Ras/Raf1 complexes were mainly located in the cell membrane, while the Raf1 control was uniformly distributed in the cytoplasm. However, the complexes of Raf1 and K-RasC185S, a K-Ras mutant which loses membrane-localization, were also able to accumulate in the cell membrane. In contrast, an apparent cytosolic distribution pattern was observed in cells co-transfected with mcerulean-Raf1 and EGFP-K-RasC185S, suggesting that the membrane localization of K-Ras/Raf1 complexes is not entirely dependent on K-Ras, and that other factors, such as the irreversible conformation formed between K-Ras and Raf1 may play a role. This study sheds light on the interaction between K-Ras and Raf1 and provides a practical method to elucidate the mechanism underlying K-Ras and Raf1 binding to the cell membrane.

## 1. Introduction

The Ras protein family is a major component of numerous cellular signaling pathways that control cell differentiation, proliferation, survival, cell cycle entry and cytoskeletal dynamics [1]. Dysregulation of these cellular functions is a hallmark of diseases including cancers. There are three major Ras family members, N-Ras, H-Ras and K-Ras, and amongst these K-Ras is found to be the most frequently mutated protein in human cancers [2]. As an important molecular switch in signal transduction [3], K-Ras interacts with various effectors to produce different responses to extracellular stimulations. For example, Raf1 is a well characterized kinase in the MAPK cascade, which proceeds through the activation of MAPK/ERK (MEK, also known as MAPKK) and extracellular signal-regulated kinases (such as ERK) [4,5]. Fusion proteins containing the K-Ras membrane localization sequence and the carboxy terminus of Raf1, which is normally cytosolic, were constitutively active in membrane [6], suggesting that Ras functions as a membrane-bound anchor for Raf1. Recently, detailed interactions between K-Ras and Raf1 have been elucidated including the specifics of the conformational change which Raf1 undergoes upon binding to K-Ras [7]. However, there are still some unresolved issues regarding their interactions such as where and how the activation of Raf1 and K-Ras occurs in cells, whether K-Ras and Raf1 simply traffic together or are part of a larger multicomponent signaling complexes, as well as whether the ultimate cellular localization of the K-Ras/Raf1 complexes is independent of the original Raf1 and K-Ras locations.

A variety of methods have been used to assess the interactions between the proteins involved in the K-Ras signaling cascade, including Western blotting [8–10], fluorescence resonance energy transfer (FRET) [11–14], two-hybrid assays [15,16], and bimolecular fluorescence complementation (BiFC) [17–19]. BiFC is based on complementation between two non-fluorescent fragments of a fluorescent protein when they are brought together by interactions between proteins fused to each fluorescent protein fragment inside living cells. Interactions of these proteins bring the two complementary non-fluorescent fragments within proximity, allowing the reporter protein to reform in its native three-dimensional structure and emit its fluorescent signal [20–22]. BiFC permits the observation of multi protein complexes formation intuitively and with high sensitivity. A key feature of BiFC is that complexes formed by fluorescent protein fragments are often irreversible, as opposed to FRET-based fluorescent protein reporter systems, where interaction between two proteins follows a dynamic interaction and thus may not be observable if the interaction is sufficiently transient. Thus, the comparison of cellular localization of proteins of interest in these two systems provides a way to observe their position changes at distinct associational states.

In the present study, the interactions of K-Ras/Raf1 and K-Ras-C185S/Raf1 (an iso form of K-Ras which has a mutation of C185 to S and is not able to bind to the membrane [23]) were examined in COS-7 cells under different conditions to probe cellular trafficking behaviors of Raf1 and K-Ras to membrane. This study provides new insights into the understanding of Raf1 binding to cell membrane and offers a practical method to elucidate the functional role of the signaling proteins in K-Ras pathways.

## 2. Experimental Section

### 2.1. Plasmids Construction

**Figure 1.**
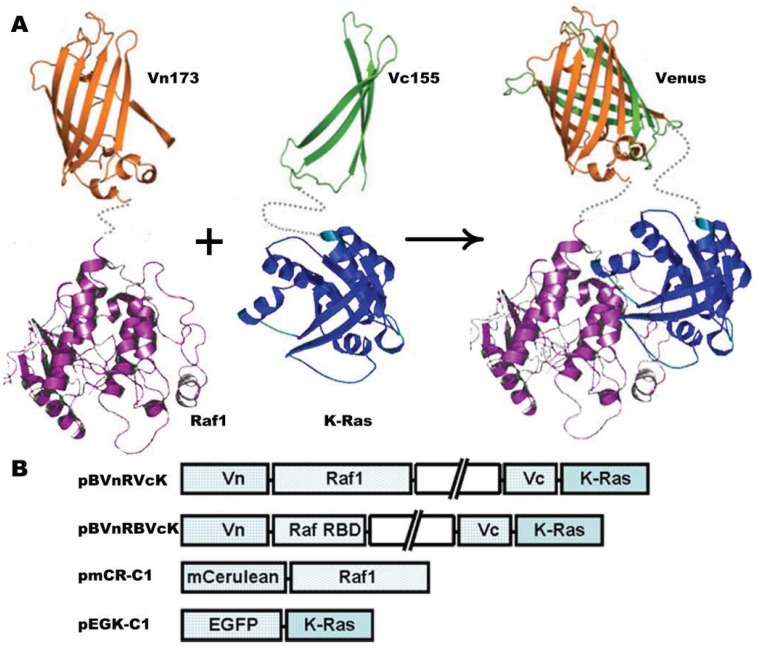
Principle of BiFC for K-Ras-Raf1. (**A**) View of BiFC principle. N- and C-terminal fragments of fluorescent proteins Venus were fused to N-termindal of Raf1 and K-Ras, respectively. The interaction between Raf1 and K-Ras brings N- and C-terminal fragments in proximity to reconstitute an intact Venus. (**B**) Schematic representation of the plasmid constructs made and used in this study.

The plasmid vector carrying K-Ras and H-Ras gene was kindly provided by Yoel Kloog [24]. Raf1 DNA was amplified following reverse transcription using polymerase chain reaction (RT-PCR) with the extraction of total RNA from HeLa cells as the templates. BiFC was carried out using the pBudCE4.1 vector (Invitrogen, Carlsbad, CA, USA) for simultaneous expression of two genes in mammalian cell lines. Venus (1–172) (Vn173) and Venus (155–238) (Vc155) were amplified by PCR and inserted into the Hind III-Sal I and Not I-Xho I sites, respectively. K-Ras was amplified using the upstream primer 5′-ATGACTGAATATAAACTTG-3′ and the downstream primer 5′-CATAATTAC ACACTTTG-3′. K-Ras mutant K-Ras-C18S was amplified using the same upstream primer and the downstream primer had a sequence of 5′-CATAATTACAGACTTTGTC-3′. K-Ras or K-Ras mutant K-Ras-C185S was subcloned into XhoI-Mlu I restriction sites downstream Vc155. Raf1 was subcloned into Sal I-BamH I sites of pBudCE4.1 vector downstream of Vn173 to generate pBud-Vn-Raf1-Vc-K-Ras (pBVnRVcK) or pBud-Vn-Raf1-Vc-K-Ras-C185S (pBVnRVcK-CS), respectively. BiFC vector pBud-Vn-RBD-Vc-K-Ras (pBVnRBVcK) was constructed by inserting Ras binding domain (RBD) of Raf (amino acids 52–132) into Sal I-BamH I sites; K-Ras was replaced with H-Ras to generate pBud-Vn-RBD-Vc-H-Ras (pBVnRBVcH). Plasmid construct pBud-Vn-Raf1-Vc (pBVnRVc) which has no genes upstream and downstream Vc155 was used as a negative control. pEGFP-K-Ras-C1(pEGK-C1) or pmCerulean-Raf1-C1(pmCR-C1) was constructed by insertion of K-Ras or Raf1 into plasmid EGFP-C1 (Clontech, Palo Alto, CA, USA)) or mCerulean-C1 [25], respectively. Schematic representation of the plasmid constructs is shown in Figure 1(B). All of the plasmid constructs were verified by sequencing.

### 2.2. Cell Culture, Transfection and Microscopy

The African green monkey kidney fibroblast-like cell line COS-7 was used as host for transfection with recombinant plasmid vectors. The cells were cultured in Dulbecco′s modified Eagle′s medium (DMEM, Gibco, Grand Island, NY, USA), supplemented with 10% fetal bovine serum (FBS, Gibco), 100 mg/mL streptomycin and 100 U/mL penicillin (Life Technologies, Inc., Carlsbad, CA, USA) at 37 °C in a humidified atmosphere with 5% CO_2_. Semi-confluent COS-7 cells were seeded into a 96-well plate at 4,000 cells/well and serum-starved by incubation in serum-free DMEM at 37 °C in a humidified atmosphere with 5% CO_2_ for 24 h before transfection.

The plasmid vectors carrying genes described above were transfected into the COS-7 cells using Lipofectamine^TM^ 2000 (Invitrogen) according to the manufacturer′s protocol. Following serum starvation for 16 h after transfection, cells were treated with epidermal growth factor (EGF) (Peprotech, Rocky Hill, NJ, USA) at a final concentration of 100 ng/mL for 5 h. The cells were imaged with an inverted fluorescence microscope (IX71, Olympus, Tokyo, Japan) and recorded with either a cooled color charge-coupled device camera (CCD, Pixera Penguin 150CL, San Jose, CA, USA), or a confocal laser scanning microscopy (FV1000, Olympus) with CO_2_ incubator (MIU-IBC, Olympus). The images were analyzed using Image J, the Java-based image processing and analysis program (Wayne Rasband, Research Services Branch, National Institute of Mental Health, Bethesda, MD, USA). To compare the efficiencies of fluorescence complementation in different cells, the cells were co-transfected with the pmCerulean-C1 expression vector. The ratio between YFP and CFP emissions was quantified for every cell expressing CFP in a field to ensure unbiased data analysis [26].

## 3. Results and Discussion

### 3.1. Characterization of the Raf1/K-Ras BiFC System

To investigate the cellular localization of K-Ras and Raf1, two sets of plasmids were constructed; (1) Vectors encoding Raf1 and K-Ras BiFC fusions, and (2) Vectors encoding Raf1 and K-Ras fused to standard fluorescent proteins (Figure 1).

EGF, which stimulates Raf1/K-Ras interaction [27], was selected to assess the reliability of the probing systems. To avoid the specific effect of EGF existing in serum, all cells in studies were serum starved for 24 h before transfection. Serum-starved cells were subsequently transfected with pBVnRVcK. EGF was added at 16 h after transfection, and the respective BiFC fluorescence was observed directly after EGF treatment with real-time imaging in COS-7 cells. The plasmids pBVnRBVcK, pBVnRBVcH and pBVnRVcKCS (see Figure 1) were separately transfected using the same protocol. In these BiFC studies, mCerulean-C1 was co-transfected as an internal reference and ratios of yellow/blue fluorescent intensity were recorded.

**Figure 2.**
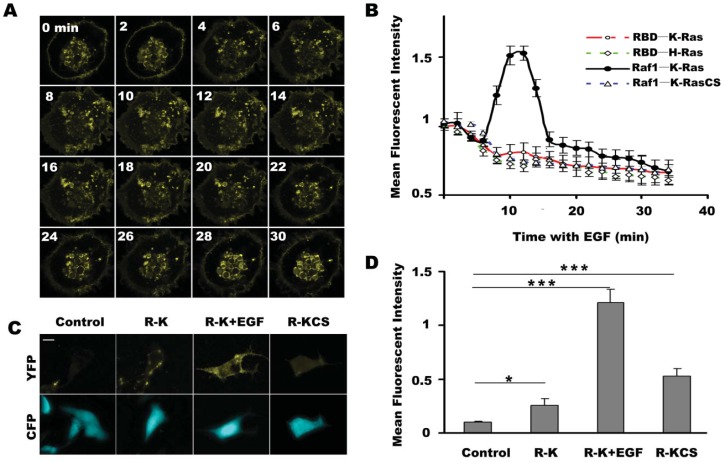
Kinetics of K-Ras/Raf1 interaction. (**A**) Serum starved COS-7 cells were transfected with VN-Raf1-VC-K-Ras, and 16 h later were treated with 100 ng/mL EGF. Time interval between adjacent two images is 2 minutes. (**B**) BiFC vectors were co-transfected with mCerulean-C1. Fluorescent intensity was represented by ratio of yellow and cyan fluorescence. Mean fluorescent intensity of RBD/K-Ras, RBD/H-Ras, Raf1/K-Ras and Raf1/K-RasC185S were monitored upon EGF stimulation. (**C**) Confocal images of cells co-transfected with pBud-Vn-Raf1-Vc-K-Ras (K-Ras 12v or K-Ras C185S) and pmCerulean-C1. (**D**) Mean fluorescent intensity of R-K (cells expressed VN-Raf1-VC-K-Ras), R-K+EGF (EGF stimulated cells expressed VN-Raf1-VC-K-Ras for 5 h) and R-KCS (cells expressed VN-Raf1-VC-K-RasC185S). In each experiment (n = 3), 50 individual cells in each group were measured. Significant differences in fluorescence ratio (* *P* < 0.05 and *** *P* < 0.001) were observed between groups as indicated. Scale bar, 10 μm.

As shown in Figure 2(A), the fluorescence signal of the pBVnRVcK group initially appeared mainly in the nucleus region and cell membrane, and gradually spread to the cytoplasm with the time after EGF stimulation, and finally returned to the initial status at 20 minutes after EGF stimulation. The quantitative results in Figure 2(B) show that BiFC fluorescence of Raf1/K-Ras reached the maximum at about 15 minutes and appeared to stabilize at 30 minutes (Figure 2(B)). In contrast, fluorescence of RBD/K-Ras, RBD/H-Ras and Raf1/KrasC185S decreased throughout the observation period.

In addition, fluorescent signal of the cell membrane in pBVnRVcK group increased following EGF stimulation (Figure 2(A)). These data shows that the Raf1/K-Ras BiFC system was sensitive to EGF stimulation.

Next, we investigated the effect of a longer EGF incubation time on the fluorescent intensity of the BiFC systems described above. Serum-starved COS-7 cells were transfected with pBVnRVc, pBVnRVcK or pBVnRVcKCS, and the pBVnRVc group was taken as a control. The effect of EGF on the BiFC signal of pBVnRVcK group was assessed in the presence or absence of EGF stimulation for 5 h. As shown in Figure 2(C), little BiFC fluorescence was detected in the control cells expressing Vn-Raf1/Vc. Upon EGF stimulation, cells expressing Vn-Raf1/Vc-K-Ras showed remarkably enhanced fluorescence signal compared to that without EGF treatment. The analyzed data in Figure 2(D) showed that the fluorescence of Vn-Raf1/K-RasC185S was about two times of Vn-Raf1/Vc-K-Ras, and the BiFC fluorescence in EGF stimulated cells was about four times of the group without EGF treatment, whereas EGF stimulation did not lead to a significant change in fluorescence signal of the cells transfected with pBVnRVcKCS (data not shown). Results above show that the BiFC system composed of Raf1/K-Ras is sensitive for EGF stimulation, and the trend of BiFC fluorescence change is consistent with previous report on the K-Ras response to EGF stimulation [27], indicating that the activation of K-Ras can promote its complex formation with Raf1, and the Raf1/K-Ras interaction in BiFC system has not been interfered by the fluorescent protein fragments they connected, and this system is suitable for further studies of Raf1/K-Ras interaction.

### 3.2. Sub-Cellular Localization of Raf1/K-Ras and Raf1/K-Ras-C185S in COS-7 Cells

**Figure 3.**
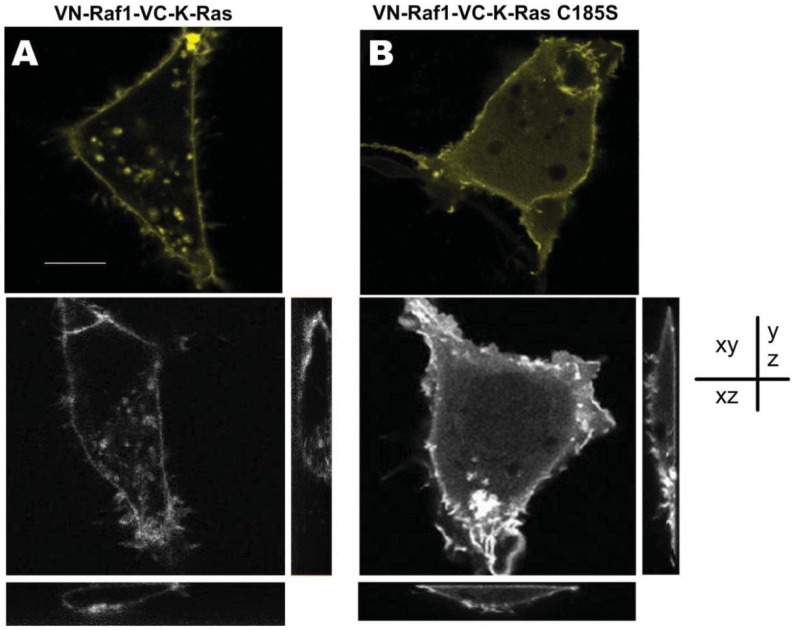
Sub-localization of Raf1/K-Ras complexes and Raf1/K-Ras-C185S in BiFC assays. (**A**) COS-7 cells expressing VN-Raf1/VC-K-Ras were observed with confocal microscopy, and the xy, xz, and yz images are shown, respectively. (**B**) COS-7 cells were transfected with expression vectors for VN-Raf1-VC-K-Ras C185S. Scale bar, 10 μm.

As a link of a variety of downstream/upstream signaling components, K-Ras has complicated and delicate relationship with Raf1 [28,29]. To investigate the intracellular localization of Raf1 and K-Ras, we transfected the COS-7 cells with two constructed BiFC vectors carrying Raf1 together with either K-Ras, or K-Ras-C185S. As shown in Figure 3, sub-cellular localization of the K-Ras/Raf1 complexes which was mainly distributed in the cell membrane (Figure 3(A)) varied from the K-Ras-C185S/Raf1, which was mainly localized in the cytoplasm (Figure 3(B)), and these distinct subcellular localizations were further confirmed in different imaging depth (Figure 3(A,B)), indicating that the C-terminal CAAX site is the determinant for sub-cellular localization of K-Ras/Raf1 complexes, which is consistent with previous reports [23]. However, with respect to membrane localization, the Raf1/K-Ras-C185S complexes could move to the cell membrane. Since the fluorescent signal observed in BiFC system only occurs in the case of protein complexes formation, our finding suggests that Raf1 and K-Ras-C185S form protein complexes in the cell membrane, and such protein complexes formation is likely to play a key role in the cellular trafficking of Raf1.

### 3.3. Subcellular Localization of Co-Expressed K-Ras and Raf1

**Figure 4.**
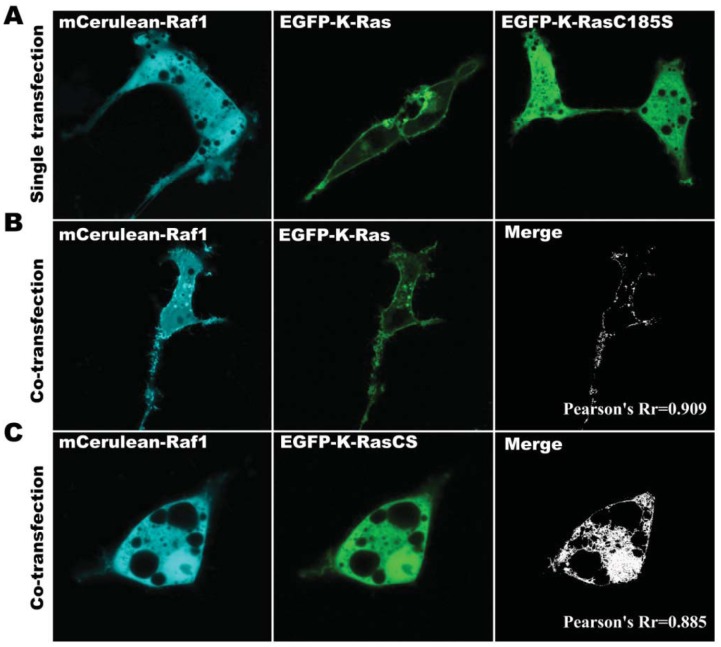
Sub-cellular localization of K-Ras, K-Ras-C185S and Raf1 in COS-7 cells. (**A**) Expression of mCerulean-Raf1, EGFP-K-Ras, and EGFP-K-Ras-C185S in COS-7 cells, respectively. (**B**) Co-expression of EGFP-K-Ras and mCerulean-Raf1 in COS-7 cells, and co-localization of the two proteins was clearly observed at the cell membrane. (**C**) Co-expression of EGFP-K-Ras-C185S and mCerulean-Raf1 in COS-7 cells. The mean colocalization Pearson′s Rr of Raf1-Kras and Raf1-K-RasC185S are listed in (B) and (C). Colocalization analysis was performed using the Image J software. Scale bar, 10 μm.

To investigate the localization of protein fusions and the role of K-Ras and Raf1 in sub-cellular localization of the complexes in living cells, the plasmid vectors carrying EGFP-K-Ras and mCerulean-Raf1 were either individually or co-transfected into COS-7 cells, and their subsequent localizations were visualized using confocal microscopy. As shown in Figure 4, when individual transfections were performed, mCerulean-Raf1 was found uniformly distributed in the cytoplasm (Figure 4(A)), whereas EGFP-K-Ras was mainly located on the cell membrane (Figure 4(B)). When EGFP-K-Ras and mCerulean-Raf1 were co-transfected into COS-7 cells, in addition to the localization in the cytoplasm, Raf1 was clearly observed in the cellular membrane, which shows distinct difference from the individual transfection result (Figure 4(B)). Moreover, significant co-localized signal between K-Ras and Raf1 was found in the cell membrane (Figure 4(B)), suggesting that K-Ras affects the cellular localization of Raf1, and the cellular interaction between K-Ras and Raf1 is likely to facilitate the membrane-binding ability of Raf1. Additionally, when K-Ras was replaced with K-Ras-C185S in the co-transfection study, both Raf1 and K-Ras-C185S lost their membrane-binding abilities (Figure 4(C)), further confirming the important role of K-Ras in Raf1 membrane localization. These data also indicate that fusing with fluorescent proteins did not change the localization of Raf1, K-Ras and K-RasC185S.

## 4. Conclusions/Outlook

Raf1 is an important effecter protein of K-Ras. Only after Raf1 is brought to the plasma membrane and further activated by Ras, does the MAPK/ERK signaling pathway start [4,5]. The objective of this study was to observe cellular localizations of Raf1 and K-Ras and further explore the mechanism of Raf1 transferring from cytoplasm to membrane organelles.

Sub-cellular localization of Raf1/K-Ras was observed before and after EGF stimulation in COS-7 cells with BiFC assay. Our findings are consistent with reported results generated from biochemical approaches [4,5,8,30]. This suggests that the Raf1/K-Ras interaction in BiFC system has not been destructed by their respectively connected fluorescent protein, and such system is well-suited for the study of Raf1/K-Ras interaction.

It is reported that the C-terminal sequence of Ras protein is pivotal for its membrane orientation. The CAAX sequence at C-terminal of K-Ras and its close upstream region with poly-L sequence function together to determine cytoplasmic membrane orientation [23]. Moreover, the CAAX sequence directs the post-translational modification (PTM) of the K-Ras carboxyl terminus and is very important for K-Ras activity [31–33]. Thus, the comparison of the effect of K-Ras and CAAX mutated K-Ras protein K-RasC185S on the cellular localization of Raf1 may provide useful information regarding the cellular interaction between K-Ras and Raf1. In this study, we compared the localization of K-Ras/Raf1 complexes, and co-expressed K-Ras and Raf1 in living COS-7 cells. In pBVnRVcK transfected cells, fluorescence mainly located in the cell membrane (Figure 3(A)), whereas in the cells co-transfected with EGFP-K-Ras and mCerulean-Raf1, green and cyan fluorescence colocalization was observed mainly on the membrane, which was similar to the Rn-Raf1/Vc-K-Ras expressed cells (Figure 4(B)). Cells expressing Vn-Raf1/K-RasC185S showed obvious membrane localized fluorescence (Figure 3(B)). However, both cyan and green fluorescence were uniformly distributed in the cytoplasm of the cells co-transfected with mCerulean-Raf1 and EGFP-K-Ras-C185S (Figure 4(C)). The main difference between the proteins interaction in BiFC assay and the co-expressed system lies in the fact that their dissociation abilities are different. Notably, the complexes in the BiFC assay are formed by two interacting proteins that cannot be dissociated [22]. When the cells were co-transfected with GFP-K-Ras and mCerulean-Raf1, only a small portion of the green and cyan fluorescent signal co-localized on the cell membrane, while most of the cyan fluorescence signal was present within the cytoplasm (Figure 4(B)); yet green or cyan fluorescence was evenly distributed throughout the cells co-transfected with GFP-K-RasC185S and mCerulean-Raf1 (Figure 4(C)). This finding is similar with previous reports on the interactions between Raf1 and K-Ras [4,15]. Moreover, we have observed in Figure 2(C) that the BiFC assay could detect a background level binding capacity between K-Ras and Raf1, and such affinity between K-RasC185S and Raf1 is even higher than that of K-Ras and Raf1. This low-level association exits in the form of transient dynamic balance and may be easily dissociated under physiological conditions. However, such dissociation will not occur in the BiFC system, leading to the accumulation and amplification of the fluorescent signals in cells. Therefore, distinct cellular localization events were found between BiFC and co-transfected cells. Additionally, only K-Ras fused with fluorescent protein fragment Vc can be detected through interacting with Vn-Raf1 fusion in BiFC assay. This would exclude the interference of endogenous K-Ras, which could also bring Raf1 to the cell membrane.

Finally, our results suggest that the combination and translocation of Raf1/K-Ras to the membrane occurs as follows: the combination between Raf1 and K-Ras on the membrane alters the conformation of Raf1/K-Ras complexes, and enhances its membrane binding capacity. Thereafter, such combination no longer relies on the anchors of K-Ras on the cell membrane. In other words, the Raf1/K-Ras complexes will not lose their positioning in the cell membrane while association occurs in the cell membrane, and unless they are separated. This may explain why a small amount of fluorescent signal was observed on the membrane after the Raf1/K-Ras-C185s complexes formation. Although the combination of Raf1/K-RasC185S in BiFC may not occur under physiological conditions, investigating this interaction under the special conditions of BiFC may provide new insights into the understanding of the cellular interaction between Raf1 and K-Ras.

In summary, the intercellular interaction of Raf1 and K-Ras was investigated using BiFC assay and a co-expression system with fluorescent protein fusions. We conclude that activation of K-Ras promotes complexes formation with Raf1. The membrane localization of Raf1 is not entirely dependent on K-Ras, whereas the K-Ras/Raf1 complexes formation plays a key role in this process.
